# The role of ultrasound before instrumental vaginal delivery may be underestimated

**DOI:** 10.1111/aogs.14458

**Published:** 2022-09-20

**Authors:** Zhengli Liu, Zhongxia Fu, Lin Yang

**Affiliations:** ^1^ Department of High‐risk Obstetrics Gansu Provincial Maternity and Child‐care Hospital Lanzhou City Gansu China; ^2^ Department of Pediatrics Gansu Provincial Maternity and Child‐care Hospital Lanzhou City Gansu China; ^3^ Perinatal Medical Center Gansu Provincial Maternity and Child‐care Hospital Lanzhou City Gansu China

Sir,

For decades, ultrasound has been used in various fields of medicine. However, because of the lack of high‐quality randomized controlled trials, its advantages over traditional digital vaginal examination in women undergoing instrumental vaginal delivery remain to be further verified. With great interest, we read the article entitled “Ultrasound vs routine care before instrumental vaginal delivery: A systematic review and meta‐analysis” published in *Acta Obstetricia et Gynecologica Scandinavica*. The authors concluded that ultrasound did not translate the accuracy of more precise fetal head position into better maternal or neonatal outcomes.^1^ We congratulate the authors for their very comprehensive work in assessing the impact of ultrasound on postpartum outcomes in patients undergoing instrumental vaginal delivery.

We would like to point out and highlight some possible flaws in the included studies. For example, in the study by Ghi et al, the authors mentioned that obstetricians involved in patient recruitment randomized only women with easy fetal extraction, and opted for systematic ultrasonography in cases deemed challenging preoperatively.^2^ Therefore, the included studies have large loopholes and deficiencies in the selection of randomized groups of patients. In other words, doctors still subconsciously believe that ultrasound has advantages in instrumental vaginal delivery, so they prefer to choose ultrasound in the case of some intractable cases. The possibility that the practical advantages of ultrasound may be obscured in this way requires further consideration and validation. In addition, the authors summarize the minimum sample size required for each study on the primary outcome. It can be seen that the sample size of most studies did not meet the minimum standard, which means that it is difficult to deny the beneficial effect of ultrasound on maternal and perinatal outcomes in instrumental vaginal delivery based on the available aggregated data.

**FIGURE 1:**
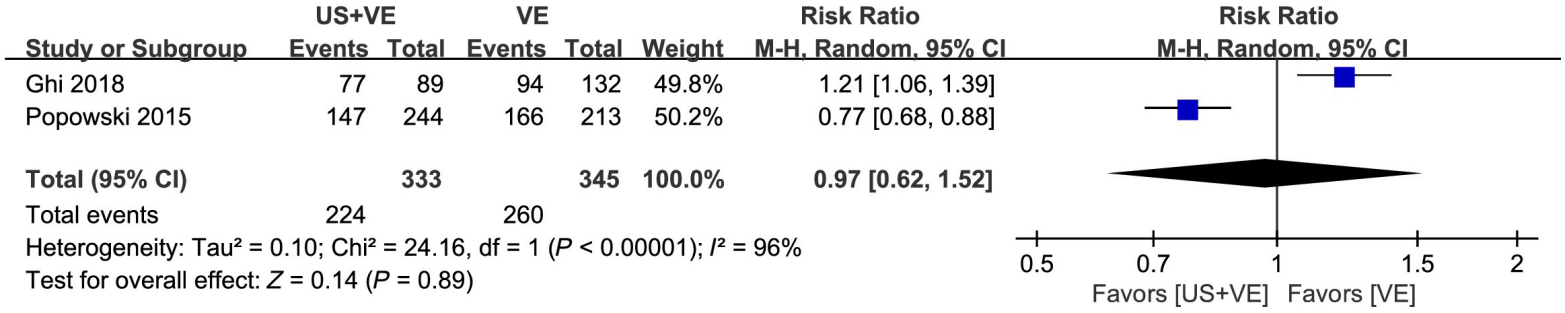
Pooled relative risks with their 95% confidence intervals for episiotomy between the two groups

The authors refer to episiotomy in the abstract, but no relevant summary data are provided in the results. Of the five included studies, only two discussed episiotomy after instrumental vaginal delivery between the two groups. Popowski et al found that the ultrasound group had a lower episiotomy rate than the non‐ultrasound group.^3^ However, the study by Ghi et al came to the opposite conclusion: the ultrasound group was associated with a higher rate of episiotomy.^2^ When the two studies were pooled, no difference in the proportion of episiotomy was found between the two groups (Figure 1). Ghi et al may attribute this diametrically opposite result to a higher probability of non‐occipital anterior findings in the ultrasound group.^2^ Therefore, the accuracy of this outcome needs to be viewed with caution.

Misdiagnosis or failure to correctly identify fetal head position is generally considered to be one of the causes of a failed instrumental delivery, which in turn contributes to increased neonatal morbidity.^4^ Although the existing evidence does not prove that ultrasound can bring more practical clinical advantages, it is limited by the shortcomings of existing clinical research design and insufficient sample size. Therefore, more rigorous randomized controlled trials with large sample sizes are needed to reveal its possible clinical advantages.
